# Current Technologies Unraveling the Significance of Post-Translational Modifications (PTMs) as Crucial Players in Neurodegeneration

**DOI:** 10.3390/biom14010118

**Published:** 2024-01-16

**Authors:** Saima Zafar, Shehzadi Irum Fatima, Matthias Schmitz, Inga Zerr

**Affiliations:** 1 Department of Neurology, Clinical Dementia Center and DZNE, University Medical Center Goettingen (UMG), Georg-August University, Robert-Koch-Str. 40, 37075 Goettingen, Germany; 2 Biomedical Engineering and Sciences Department, School of Mechanical and Manufacturing Engineering (SMME), National University of Sciences and Technology (NUST), Bolan Road, H-12, Islamabad 44000, Pakistan

**Keywords:** PTM, Alzheimer’s disease, Parkinson’s disease, proteomics, tau phosphorylation, synuclein, neuronal dysfunction

## Abstract

Neurodegenerative disorders, such as Parkinson’s disease, Alzheimer’s disease, and Huntington’s disease, are identified and characterized by the progressive loss of neurons and neuronal dysfunction, resulting in cognitive and motor impairment. Recent research has shown the importance of PTMs, such as phosphorylation, acetylation, methylation, ubiquitination, sumoylation, nitration, truncation, O-GlcNAcylation, and hydroxylation, in the progression of neurodegenerative disorders. PTMs can alter protein structure and function, affecting protein stability, localization, interactions, and enzymatic activity. Aberrant PTMs can lead to protein misfolding and aggregation, impaired degradation, and clearance, and ultimately, to neuronal dysfunction and death. The main objective of this review is to provide an overview of the PTMs involved in neurodegeneration, their underlying mechanisms, methods to isolate PTMs, and the potential therapeutic targets for these disorders. The PTMs discussed in this article include tau phosphorylation, α-synuclein and Huntingtin ubiquitination, histone acetylation and methylation, and RNA modifications. Understanding the role of PTMs in neurodegenerative diseases may provide new therapeutic strategies for these devastating disorders.

## 1. Introduction

Among all the biological compounds, proteins show the most variations in structural and functional properties. In the cellular environment, proteins are synthesized by the process of translation. The amino acid side chains of protein undergo different chemical changes during or after biosynthesis. It involves enzyme-facilitated covalent processing events that add or proteolytically cleave modifying groups to a protein. For instance, certain examples of the modifying group may be acetyl, glycosyl, phosphoryl, and methyl, up to single or multiple amino acid chains [1]. These alterations are termed as post-translational modifications (PTMs) [2] and are crucial factors of non-functional protein aggregation in the pathogenesis of neurodegeneration [1,2].

Any modification in the PTMs or linked mechanisms increases the aggregation or accumulation of misfolded protein, the neuronal dysfunction. PTMs can be reversible or permanent. The reversible responses comprise covalent modifications, and the permanent ones, which continue as an irreversible response, comprise proteolytic modifications. PTMs can occur on individual amino acids or multiple amino acids, resulting in significant alterations to the biochemical properties at the modified sites [1,2].

These modifications significantly progress the complexity and are responsible for the extent of the modification between the variability of the proteins encoded in the genome and their physiological functions [2]. PTMs are observed in numerous cellular organelles including the nucleus, cytoplasm, Golgi apparatus, and endoplasmic reticulum and are involved in various functional regulation such as secretory proteins, membrane proteins and histones [1], molecular trafficking, protein lifespan, protein–protein interactions, protein solubility, the regulation of metabolism and defense processes, enzyme function and assembly, cell–matrix interactions, receptor activation, protein folding, protein localization, and in cellular recognition events and morphological alterations in the respective level [3,4,5]. These modifications greatly increase complexity and dramatically expand the encoded flexibility of a living system [6,7,8,9,10,11,12,13].

## 2. Biogenesis of PTMS

The biogenesis of PTMs can occur at various phases of the life cycle of protein during synthesis, folding, trafficking, and degradation. Some PTMs are added co-translationally, while others are added post-translationally, often in a specific order or sequence. The order and timing of PTMs can affect the protein’s function and activity, as well as its interaction with other proteins and cellular components [7,8,9,10,11,12,13,14]. As mentioned earlier, PTMs are a crucial aspect of protein biogenesis, and they play important roles in many cellular processes, including signaling, metabolism, and gene expression [15]. PTMs can occur at different sites on the protein molecule, including amino acids such as serine, threonine, tyrosine, lysine, and arginine [15,16]. The specific type and site of PTM can greatly affect the function, stability, and localization of the protein, as well as its interactions with other molecules [16].

The biogenesis of PTMs can occur at different stages of protein synthesis and processing, and the specific mechanisms and enzymes involved can vary depending on the type of PTM. For example, some PTMs are added co-translationally, meaning they occur while the protein is still being synthesized by the ribosome [14]. This can involve the coordinated action of several enzymes, such as chaperones, signal peptidases, and modifying enzymes. In contrast, other PTMs are added post-translationally, after the protein has been fully synthesized and folded [17,18,19,20]. This can involve the action of specific modifying enzymes, such as kinases, phosphatases, acetyltransferases, and glycosyltransferases, which recognize and modify specific sites on the protein molecule [1,21].

The biogenesis of PTMs can also be regulated by various signaling pathways and cellular processes. For example, many PTMs are regulated by protein kinases and phosphatases, which can be activated or inhibited in response to extracellular signals, such as hormones or growth factors [22]. Other PTMs can be regulated by the availability of substrate molecules, such as sugars or lipids, which can vary depending on the metabolic state of the cell, i.e., induction of mitochondrial and lysosomal biogenesis by adenosine monophosphate-activated protein kinase phosphorylation of folliculin-interacting protein 1(FNIP1) [23]. Additionally, the biogenesis of PTMs can be influenced by other factors, such as protein–protein interactions, cellular localization, and protein turnover [21,24].

Therefore, the biogenesis of post-translationally modified proteins is termed as a complex and dynamic process that involves a variety of mechanisms and enzymes [25]. The specific type and site of PTM can greatly affect the function and activity of the protein and can be regulated by a variety of signaling pathways and cellular processes [24,26].

## 3. Types of PTMS

**Figure 1:**
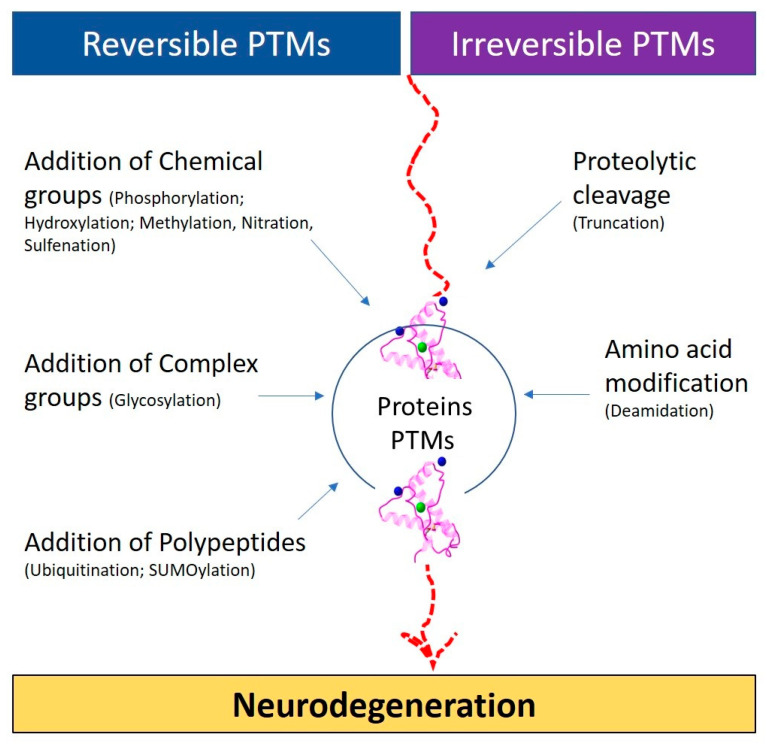
Types of post-translational modifications (PTMs).

Around 400 different types of PTMs affect many aspects of protein functions [1] and these PTMs can be categorized into five main groups (Figure 1) [1].

3.1 Addition of chemical groups (phosphorylation; hydroxylation; methylation, nitration, sulfenation);
3.2 Addition of complex groups (glycosylation);
3.3 Addition of polypeptides (ubiquitination; SUMOylation);
3.4 Proteolytic cleavage (truncation);
3.5 Deamidation.

We can refer to PTMs as critical alterations that regulate protein function and cellular processes [2,27]. The diversity of PTMs and their regulation by specific enzymes and signaling pathways emphasize their importance in maintaining cellular homeostasis and responding to environmental cues.

### 3.1. Addition of Chemical Groups (Phosphorylation; Hydroxylation; Methylation, Nitration, Sulfenation)

#### 3.1.1. Phosphorylation

Phosphorylation is a type of PTMs that plays a vital role in regulating protein localization, activity, and interactive association with other molecules. This modification comprises the addition of a phosphate group to the hydroxyl group of serine, threonine, or tyrosine amino acids which can alter the conformation, charge, and function of the protein [14]. The phosphate group is covalently linked to the amino acid residue by a phosphoester bond formed by a kinase enzyme, while the reverse reaction is catalyzed by phosphatases [28,29].

Phosphorylation is involved in several processes at the cellular level, including cell signaling, cell cycle regulation, apoptosis, and metabolism [2,30], and dysregulation can lead to several diseases, such as neurodegenerative disorders and diabetes [2,31]. Phosphorylation can induce several changes in protein function, including the activation or inhibition of enzymatic activity, the promotion or prevention of interactions between proteins, and the modulation of protein stability or degradation [2]. The phosphorylation of specific serine residues in the transcription factor CREB (cAMP-response element-binding protein) enhances its interaction with the coactivator CBP (CREB-binding protein) and promotes gene expression [32,33,34]. Similarly, the phosphorylation of residues of serine and threonine in the protein kinase AKT (protein kinase B) activates its kinase activity and promotes cell survival and growth [28,29].

Phosphorylation can also regulate protein localization by affecting their affinity for cellular compartments or transporters. For instance, the phosphorylation of serine residues in the tau protein can modulate its binding to microtubules and promote its translocation from the axon to the somatodendritic compartment of neurons [35]. Furthermore, phosphorylation can act as a signal for protein degradation by targeting proteins for recognition and degradation by the lysosome or proteasome pathways [16] and these highly phosphorylated and soluble forms of tau are additionally linked with altered synaptic function and cellular loss [36].

#### 3.1.2. Hydroxylation

Hydroxylation is a common type PTM in which hydroxyl groups are added to various amino acid residues in proteins. Proline, lysine, and asparagine are examples of such residues that can undergo hydroxylation [37,38]. Hydroxylases, a family of enzymes that rely on molecular oxygen and cofactors such as Fe^2^⁺ and α-ketoglutarate, catalyze this process [39].

Proline hydroxylation is crucial for stabilizing the collagen triple helix, while lysine hydroxylation regulates gene expression and collagen cross-linking in bone and cartilage [40,41]. The hydroxylation of lysine residues has also been shown to have important biological functions, such as the regulation of gene expression through histone hydroxylation and collagen cross-linking in bone and cartilage [42]. A few proteins have also been found to undergo the hydroxylation of asparagine residues, affecting their stability and function. Although the hydroxylation of tryptophan and tyrosine has been reported in some proteins, its biological significance remains unclear. The dysregulation of hydroxylation has been linked to several disorders, including neurodegeneration, bone disorders and also modification of the Notch signaling dynamics [43,44,45]. Therefore, understanding the biological roles and regulatory mechanisms of hydroxylation is vital for developing therapies to treat related diseases.

#### 3.1.3. Methylation

Methylation has a significant role in the regulation of gene expression and protein function [46]. This modification is the addition of a methyl group (CH₃) to amino acids, particularly arginine and lysine, or the N terminus of proteins [47].

The addition of methyl groups is catalyzed by enzymes called protein methyltransferases (PMTs) and removed by protein demethylases (PDMs) [48]. Methylation can affect protein function by altering protein–protein interactions, DNA binding, and enzymatic activity [49].

Methylation can occur in both histones and non-histone proteins. In histones, methylation is frequently observed on the lysine residues of the N-terminal tail, which affects the chromatin structure and gene expression [48]. In contrast, the methylation of arginine residues in histones can either activate or repress transcription, depending on the specific residue that is modified [50]. In non-histone proteins, methylation can regulate protein–protein interactions and enzymatic activity [51]. For example, the methylation of the tumor suppressor protein p53 regulates its stability and activity [51,52,53], and linked to various other diseases, including neurodegenerative disorders. In neurodegenerative disorders such as Alzheimer’s disease (AD), the methylation of the tau protein has been found to affect its aggregation and toxicity and showed as a crucial controller of tau aggregation and neuronal health [54,55].

#### 3.1.4. Nitration

Nitration is a PTM process that can have significant effects on protein structure and function, in which a nitrogen atom is added to a protein or peptide. This modification consists of covalent attachment of a nitro group (NO₂) to tyrosine residues on a protein, which can lead to changes in the protein structure, stability, and in a variety of physiological and pathological events, including inflammation, oxidative stress, and neurodegeneration [56].

One of the mechanisms for nitration involves the reaction of tyrosine residues with nitric oxide (NO) and superoxide anion (O_2_), leading to the formation of peroxynitrite (ONOO). This highly reactive compound can then react with tyrosine residues, resulting in the addition of a nitro group to the aromatic ring [57]. Nitration can also occur through other mechanisms, including reaction with nitrating agents such as nitric acid or nitrogen dioxide [57].

Nitration has been implicated in many diseases, including Parkinson’s disease, AD, and multiple sclerosis [58]. In Parkinson’s disease, the nitration of alpha-synuclein, a protein found in Lewy bodies, has been shown to increase its toxicity and aggregation [59,60,61,62]. In AD, the nitration of the tau protein has been found to increase its aggregation and promote the formation of neurofibrillary tangles [63,64]. In multiple sclerosis, the nitration of myelin basic protein has been found to contribute to demyelination and axonal damage [65,66].

#### 3.1.5. Sulfenation

Sulfenation is a reversible PTM that involves the covalent addition of a sulfur atom to the adjacent chain of a cysteine residue in a protein, subsequent to the development of a sulfenic acid (SOH) group [67,68]. Sulfenic acid is a highly reactive species that can undergo further reactions, such as oxidation or reduction, leading to the formation of sulfinic and sulfonic acid derivatives, respectively [69]. Sulfenation plays important roles in physiological and pathophysiological mechanism and the dysregulation of this PTM has been associated with numerous diseases, including neurodegenerative disorders [70,71]. Sulfenation can occur spontaneously under oxidative stress conditions or can be catalyzed by enzymes, such as the members of the peroxiredoxin (Prx) family, which have been shown to act as redox sensors and regulators of cellular signaling pathways [72,73]. Prx2-mediated sulfenation of the transcription factor STAT3 was shown to enhance its DNA binding activity and promote cancer cell proliferation [74,75]. In addition, the sulfenation of the tumor suppressor protein PTEN by the redox-sensitive enzyme, PTEN oxidation 1 (PTENox1), was found to inhibit its phosphatase activity and promote tumorigenesis [76]. Similarly, the sulfenation of the cysteine residue in the calcium-sensing receptor (CaSR) was shown to promote its interaction with the scaffold protein filamin A and enhance CaSR-mediated signaling [77].

Several methods have been developed to detect sulfenation in proteins, including the use of specific antibodies, fluorescent probes, and mass spectrometry-based approaches [78,79]. However, the identification of specific sulfenation sites and their functional consequences remains a challenge, and further studies are needed to explicate the mechanisms underlying sulfenation-mediated protein regulation [80].

### 3.2. Addition of Complex Groups (O-GlcNAcylation)

#### O-GlcNAcylation

O-GlcNAcylation, a modification process in multicellular eukaryotes, occurs either co-transcriptionally or post-translationally in nuclear, cytoplasmic, and mitochondrial proteins. More than 5000 O-GlcNAcylated proteins have been identified in humans, indicating its involvement in adaptive cellular functions like the regulation of the cell cycle, protein interactions, signal transduction, and proteostasis [81,82]. O-GlcNAcylation includes the addition of N-acetylglucosamine (GlcNAc) to serine or threonine residues of proteins [81,82,83,84]. The O-GlcNAcylation process is facilitated by two enzymes, O-GlcNAcase (OGA) and GlcNAc transferase (OGT), which add or remove GlcNAc from target proteins, respectively [85,86]. OGT is a single polypeptide with two distinct domains, an N-terminal catalytic domain that transfers GlcNAc to substrates and a C-terminal domain that recognizes and binds target proteins [87]. OGA, on the other hand, is a cytoplasmic enzyme that hydrolyzes GlcNAc from modified proteins, allowing the cycling of O-GlcNAc modifications [88]. O-GlcNAcylation is a highly dynamic process, and its levels are regulated by various factors, including nutrient availability, hormonal signals, and cellular stress [89].

Several studies reveal that O-GlcNAcylation is a prevalent response mechanism to diverse stressors, such as heat shock, hydrogen peroxide, ammonia, lipopolysaccharide, and hypoxia-reoxygenation. Studies on different stress models indicate an overall upregulation in O-GlcNAc levels as a protective response to injury [90,91,92]. However, in some studies, a reduction in O-GlcNAc levels was observed, while others displayed complex dynamics in O-GlcNAc cycling. Additionally, in certain models, global increases in the levels of O-GlcNAc were observed, but protein-specific analysis revealed nuanced results, with some proteins demonstrating increased O-GlcNAcylation, while some showed O-GlcNAc loss [93,94,95].

### 3.3. Addition of Polypeptides (Ubiquitination; SUMOylation)

#### 3.3.1. Ubiquitination

Ubiquitination involves the attachment of the small protein, ubiquitin, to other proteins, targeting them for degradation or altering their functions. This process is regulated by a cascade of enzymes that include E1 (ubiquitin-activating enzyme), E2 (ubiquitin-conjugating enzyme), and E3 (ubiquitin ligase) [96]. The process contains the development of an isopeptide bond among the C-terminal glycine residue of ubiquitin and the lysine residue of the target protein, which can result in mono-ubiquitination, multi-ubiquitination, or poly-ubiquitination [97,98]. Additionally, ubiquitination is involved in regulating several cellular processes, including transcriptional regulation, DNA repair, protein degradation, endocytosis, and signal transduction [97].

The regulation of ubiquitination is complex, and several factors can influence its efficiency, including the structure of the target protein, the availability of ubiquitin, and the activity of E3 ligases [97]. Moreover, the modification of ubiquitin itself can also impact the efficiency of the ubiquitination process. For instance, the phosphorylation of ubiquitin can inhibit ubiquitination, while deubiquitinating enzymes (DUBs) can remove ubiquitin from modified proteins, thereby regulating the stability and activity of target proteins [99].

Therefore, ubiquitination can be defined as a critical PTM that plays a pivotal role in supporting the homeostasis at cellular level by regulating protein degradation and altering protein functions. Modification of ubiquitination has been linked to various diseases, and further research into the mechanisms underlying this process may offer new therapeutic strategies for these conditions [100].

#### 3.3.2. SUMOylation

SUMOylation comprises the covalent association of small ubiquitin-like modifier (SUMO) proteins to the target protein. This PTM is recognized to control a variety of cellular mechanisms, including gene expression, DNA repair, protein stability, and signal transduction pathways [100]. The SUMOylation process comprises the conjugation of SUMO to lysine residues on the target protein through an isopeptide bond. The SUMO proteins are synthesized as precursors, which are processed by SUMO proteases to expose the C-terminal diglycine motif that is compulsory for conjugation [99,101,102,103]. The SUMOylation of target proteins is reversible, and the elimination of SUMO is carried out by SUMO-specific proteases (SENPs), which cleave the isopeptide bond [104].

The process of SUMOylation is carried out by the cascade of an enzymatic reaction, which includes the sequential action of activating enzyme E1, conjugating enzyme E2, and ligase E3. SUMOylation plays an important role in maintaining the proper functioning of cells, and alterations in this process have been implicated in various diseases such as neurodegenerative disorders such as AD, Parkinson’s disease, and Huntington’s disease [101,102,103].

One of the well-known functions of SUMOylation is its involvement during transcriptional activity. The SUMOylation of transcription factors can either activate or repress their activity, depending on the context and the specific protein involved. For example, the SUMOylation of the tumor suppressor p53 can enhance its transcriptional activity, whereas the SUMOylation of the transcription factor Sp3 can repress its activity [105]. Another important function of SUMOylation is in the regulation of DNA repair pathways. SUMOylation is also involved in the facilitating repair proteins to the sites of DNA damage and in the assembly of protein complexes involved in DNA repair [106]. SUMOylation is a critical PTM that plays a vital function during cellular processes by a cascade of enzymes, and the modification is reversible, allowing for the dynamic regulation of protein function [106]. A deeper understanding of SUMOylation is necessary to develop novel therapies for various diseases.

### 3.4. Proteolytic Cleavage (Truncation)

Truncation is a common type of PTM that occurs in various biological contexts, such as during protein maturation, degradation, and activation. Many secreted proteins require proteolytic cleavage to remove their signal peptide or propeptide to become functional [107,108]. Additionally, some receptors and enzymes undergo cleavage to generate different isoforms with distinct functions or regulation. Caspase-8 stands as the foremost protease in the caspase activation cascade [109]. Through proteolytic cleavage, it triggers the activation of caspase-3 [110]. Notably, investigations in the brains of Alzheimer’s disease (AD) patients reveal the abundant presence of the active form of caspase-8 specifically within neurons bearing neurofibrillary tangles (NFTs) [111].

Studies have shown that truncation can modulate protein interactions with other molecules by exposing or masking specific binding domains [112]. For instance, the cleavage of the extracellular domain of the transmembrane receptor Notch by ADAM metalloproteases can activate its downstream signaling pathway by exposing its intracellular domain, which translocates to the nucleus and regulates gene expression [113]. Similarly, the proteolytic cleavage of insulin-like-growth factor binding protein 4 (IGFBP-4) by pregnancy-associated plasma protein-A (PAPP-A) releases active insulin-like growth factor 1 (IGF-1) and promotes cell proliferation and survival [114,115].

Truncation can also affect protein stability and turnover by removing destabilizing regions or exposing new degradation signals. For example, the removal of the C-terminal domain of p53 by caspases increases its stability and prevents its degradation by the ubiquitin-proteasome system [116]. On the other hand, the truncation of the RNA-binding protein TDP-43 by calpain proteases generates a C-terminal fragment that is more prone to aggregation and toxicity in frontotemporal dementia and amyotrophic lateral sclerosis [117].

Truncation has been demonstrated as a critical type of PTM that can modulate protein function, stability, and turnover by altering protein interactions, localization, and degradation. Its physiological and pathological roles have been extensively studied, providing insights into the mechanisms of cellular signaling, metabolism, and disease.

### 3.5. Deamidation

Deamidation is the process by which a glutamine or asparagine residue is transformed into another functional group. Specifically, asparagine is modified to aspartic acid or isoaspartic acid, whereas glutamine becomes either glutamic acid or pyroglutamic acid. This alteration has the potential to modify the structure, stability, and function of the protein [118].

The deamidation of the residue Asn is an automatic degradation process in proteins, leading to the creation of detrimental isoaspartate (isoAsp). Elevated levels of isoAsp within proteins result in a concomitant decline in their native structure and solubility, ultimately precipitating protein aggregation [118]. In turn, this ultimately triggers the progress of numerous diseases, including neurodegenerative diseases (NDDs) [119,120,121,122,123,124].

A recent study discusses the emerging role of blood protein deamidation not only in AD but also in other NDDs. Deamidation biomarkers, such as the levels of isoAsp and anti-aHSA IgGs, as well as the IsoAsp/IgG ratio, showed promising performance in early stages of mental decline and were significantly correlated with mental scores [125]. A larger study is required to further investigate the potential of these biomarkers in early NDD diagnostics. Additionally, more types of NDDs should be examined to fully understand the specificity of deamidation biomarkers in NDD diagnostics.

## 4. Current Technologies to Decipher PTMs

Overall, deciphering PTMs is a complex task that requires the integration of multiple techniques and approaches. The advances in MS-based proteomics, antibody-based methods, chemical labelling, bioinformatics, X-ray crystallography, NMR spectroscopy, and top-down proteomics have significantly enhanced our understanding of PTMs and their roles in protein biology and disease mechanisms (Table 1). Further exploration of PTMs using emerging technologies will likely uncover new insights into protein function and facilitate the development of new therapies for different diseases.

### 4.1. Methylation

Methylation is the addition of a methyl group to a substrate molecule and is a common PTM that plays a significant role in regulating gene expression, protein function, and cellular processes [126]. Various methods are employed to study methylation, each with its unique advantages and limitations [126].

#### Mass Spectrometry (MS)

Mass spectrometry (MS) is a pivotal analytical technique in the study of post-translational modifications (PTMs), particularly methylation, in proteins. This method has undergone significant advancements, making it indispensable in proteomics and molecular biology research [127].

The first step involves preparing the protein sample. Proteins are typically digested into smaller peptides using enzymes like trypsin, which cleaves proteins at specific amino acid residues. This digestion is crucial for simplifying the complex mixture of proteins and making them amenable to MS analysis [127]. The digested peptides are then ionized, a process that imparts a charge to these molecules [127]. The most common ionization techniques used in MS for studying methylation are electrospray ionization (ESI) and matrix-assisted laser desorption/ionization (MALDI). ESI works by applying a high voltage to a liquid sample to create an aerosol, while MALDI involves co-crystallizing the sample with a matrix material and then ionizing it using a laser [128]. Once ionized, the peptides are introduced into the mass spectrometer. Here, they are separated based on their mass-to-charge (*m*/*z*) ratio. Time-of-flight (TOF), orbitrap, and quadrupole analyzers are among the common types of mass analyzers used [128]. Each of these analyzers works based on different principles to separate and measure the *m*/*z* ratio of the peptides [128]. The separated ions are detected, and the data is recorded as a spectrum showing the intensity of the ions against their *m*/*z* ratio [127]. Advanced software is then used to analyze this data, identifying peptide masses and deducing the protein sequences. Modifications like methylation alter the mass of peptides, which can be detected as shifts in the *m*/*z* ratio in the spectrum [127]. Quantitative MS, such as tandem mass tags (TMT) or stable isotope labeling by amino acids in cell culture (SILAC), can be used to quantify methylation changes under different conditions. The validation of identified methylation sites is often performed using targeted MS techniques like Selected Reaction Monitoring (SRM) [129].

### 4.2. Phosphorylation

#### 4.2.1. Proximity Ligation Assay (PLA)

The proximity ligation assay is a sophisticated technique used for detecting and quantifying protein phosphorylation at the cellular level, offering insights into the spatial and temporal dynamics of phosphorylation events [130].

First, specific antibodies are used to target the protein of interest, with at least one antibody recognizing the phosphorylated form [130]. These antibodies are conjugated to unique DNA oligonucleotides. When the target protein is phosphorylated, the conjugated antibodies bind in close proximity, allowing the oligonucleotides to be ligated [131]. The ligated DNA is then amplified via PCR, and the amplified product is detected using fluorescence microscopy. This enables the visualization of phosphorylation events at the single-molecule level within cells [130].

#### 4.2.2. Phosphoproteomics

Phosphoproteomics is a mass spectrometry-based approach that allows for the comprehensive analysis of phosphorylation on a proteome-wide scale [131].

Proteins are extracted and digested into peptides, typically using trypsin. Specialized techniques like IMAC or MOAC are used to enrich phosphopeptides, as phosphorylated peptides are usually less abundant than their non-phosphorylated counterparts [131]. The enriched phosphopeptides are analyzed using advanced mass spectrometry techniques, such as LC-MS/MS, to identify and quantify phosphorylation sites [130]. Sophisticated bioinformatics tools are employed to process the mass spectrometry data, identifying phosphorylation sites and quantifying changes in phosphorylation under different experimental conditions [132].

### 4.3. SUMOylation

#### 4.3.1. SUMOylation Site Identification by Mass Spectrometry (SIMS)

SIMS is a mass spectrometry-based approach specifically designed to identify and characterize SUMOylation sites on proteins [133]. Proteins are extracted from cells or tissues under conditions that preserve SUMO conjugates [133].

SUMOylated proteins are enriched using SUMO-specific antibodies or affinity tags. This step often involves the use of tandem affinity purification tags to enhance specificity [134]. The enriched SUMOylated proteins are then digested into peptides, typically using enzymes like trypsin [134]. The resulting peptides are analyzed using liquid chromatography-tandem mass spectrometry (LC-MS/MS) [133]. This analysis identifies peptides that contain SUMOylation sites and characterizes the specific lysine residues modified by SUMO. The next step is the mass spectrometry analysis. The mass spectrometry data is processed using bioinformatics tools to map the SUMOylation sites and understand their functions [134].

#### 4.3.2. SUMO Protease Protection Assay (SuPrPA)

SuPrPA is a biochemical assay used to study SUMOylation dynamics and to identify SUMO-modified substrates [134].

Cells are lysed, and proteins are extracted in a buffer that preserves SUMO conjugates. The protein extract is treated with SUMO-specific proteases under controlled conditions [133]. This step results in the cleavage of SUMO from its conjugated proteins. A portion of the extract is protected from protease treatment, serving as a control. This allows for the comparison between the SUMOylated and deSUMOylated states of proteins. [134]. The treated and untreated samples are then subjected to SDS-PAGE, followed by Western blotting using antibodies against the protein of interest. This allows for the detection of shifts in molecular weight corresponding to the SUMOylation and deSUMOylation states [133]. The differences in mobility on SDS-PAGE gels between treated and untreated samples indicate the presence and extent of SUMOylation on the target proteins [135].

### 4.4. Ubiquitination

#### 4.4.1. Ubiquitin Enrichment

Ubiquitin Enrichment is a technique used to isolate and study ubiquitinated proteins from complex biological samples [135]. Cells or tissues are lysed, and proteins are extracted in a buffer that preserves ubiquitin conjugates [135]. Ubiquitinated proteins are enriched from the protein extract using antibodies that specifically recognize ubiquitin [136]. This step often involves immunoprecipitation or affinity chromatography using ubiquitin-specific antibodies or ubiquitin-binding domains (UBDs) [136]. The bound proteins are eluted from the antibody or affinity matrix under conditions that disrupt the antibody-protein interaction but preserve the ubiquitin-protein conjugates [137]. The enriched ubiquitinated proteins are then analyzed by mass spectrometry, typically using LC-MS/MS, to identify ubiquitination sites and characterize the types of ubiquitin linkages [137]. The mass spectrometry data is processed using bioinformatics tools to map ubiquitination sites and understand their functional implications [136].

#### 4.4.2. Tandem Ubiquitin Binding Entities (TUBEs)

TUBEs are engineered tools designed to bind and protect polyubiquitinated proteins, thereby facilitating their study [137]. Similar to the ubiquitin enrichment method, cells or tissues are lysed, and proteins are extracted. TUBEs, which consist of tandem repeats of ubiquitin-binding domains, are added to the protein extract [137]. They have a high affinity for polyubiquitinated chains and can protect ubiquitinated proteins from deubiquitinating enzymes (DUBs). The TUBE-bound ubiquitinated proteins are pulled down using affinity purification techniques. The ubiquitinated proteins are eluted and can be analyzed by Western blotting for specific proteins or subjected to mass spectrometry for a broader analysis of ubiquitination sites and patterns [137]. For a comprehensive analysis, the eluted proteins can be analyzed by mass spectrometry, followed by bioinformatics processing to identify ubiquitination sites and study the ubiquitin landscape [135].

### 4.5. Nitration

#### 4.5.1. Immunoprecipitation Combined with Mass Spectrometry (IP-MS)

IP-MS is a powerful technique for identifying and characterizing nitrated proteins [138]. Immunoprecipitation combined with mass spectrometry (IP-MS) is a pivotal technique for the identification and characterization of nitrated proteins [138]. Proteins are extracted from cells or tissues under conditions that preserve nitration modifications. Antibodies specific to nitrated tyrosine residues are used to selectively enrich nitrated proteins from the protein extract [139]. This step often involves the use of beads coated with anti-nitrotyrosine antibodies to capture nitrated proteins [140]. The nitrated proteins are eluted from the antibody-coated beads under conditions that disrupt the antibody-protein interaction. The eluted proteins are then digested into peptides and analyzed by mass spectrometry, typically using LC-MS/MS, to identify nitrated peptides and their specific nitration sites [141]. The mass spectrometry data is processed using bioinformatics tools to map nitration sites and understand their functional implications [141].

#### 4.5.2. Liquid Chromatography-Tandem Mass Spectrometry (LC-MS/MS)

LC-MS/MS is a highly sensitive and specific method for the analysis of protein nitration [142].

Proteins are extracted and digested into peptides, typically using trypsin. Nitrated peptides are enriched using chromatographic techniques [142]. This may involve the use of affinity columns that specifically bind to nitrated residues [142]. The enriched peptides are separated using liquid chromatography, which helps in resolving complex peptide mixtures [143]. The peptides are then analyzed by tandem mass spectrometry. This analysis provides detailed information about the mass/charge ratio of the peptides and their fragments, allowing for the identification of nitrated residues [128]. The data obtained from LC-MS/MS is analyzed using bioinformatics tools to identify and quantify nitration sites across the proteome [127].

### 4.6. Truncation

#### 4.6.1. N-Terminal COFRADIC (Combined Fractional Diagonal Chromatography)

N-Terminal COFRADIC is a sophisticated technique designed to analyze the N-termini of proteins, thereby identifying truncation events [144].

Proteins are extracted from cells or tissues and digested into peptides. The N-termini of peptides are chemically modified to differentiate them from internal peptides [144]. This modification is typically achieved through acetylation or other specific labeling techniques. The peptide mixture is fractionated using chromatographic techniques, separating peptides based on their chemical properties [144]. A unique aspect of COFRADIC is the use of diagonal chromatography, where peptides are separated in two dimensions based on their modified and unmodified states. This step is crucial for isolating N-terminal peptides. The isolated N-terminal peptides are analyzed using mass spectrometry, typically LC-MS/MS, to identify the sequences of the N-termini and detect any truncation events [145].

#### 4.6.2. C-Terminal Sequencing

C-Terminal Sequencing is a method used to analyze the C-termini of proteins, identifying truncation and other modifications at the C-terminal end. Similar to N-Terminal COFRADIC, proteins are extracted and digested into peptides [144]. The C-terminal peptides are enriched using specific chemical or enzymatic methods. This may involve the use of carboxypeptidases to selectively trim peptides, leaving only the C-termini intact [145]. The C-termini are often chemically labeled to facilitate their detection and analysis. The enriched and labeled C-terminal peptides are analyzed using mass spectrometry. This analysis identifies the sequences of the C-termini and detects truncation events [128]. The mass spectrometry data is processed using bioinformatics tools to map the C-terminal sequences and understand the implications of truncation events [143].

### 4.7. O-GlcNAcylation

#### 4.7.1. Chemoenzymatic Labeling

Chemoenzymatic labeling is a sensitive and specific method for detecting O-GlcNAc-modified proteins [145].

Proteins are extracted from cells or tissues under conditions that preserve O-GlcNAc modifications. A two-step enzymatic process is used for labeling. Initially, the O-GlcNAc residues on proteins are enzymatically labeled with a bioorthogonal functional group, such as an azide or alkyne [146]. This is typically achieved using a mutant O-GlcNAc transferase that transfers a modified UDP-GlcNAc (bearing the bioorthogonal group) to proteins [145]. The labeled proteins are then subjected to a bioorthogonal reaction (such as click chemistry) to attach a reporter tag, like a fluorophore or biotin, to the bioorthogonal group [146]. The tagged proteins can be detected and analyzed using methods like Western blotting, mass spectrometry, or fluorescence microscopy, depending on the reporter tag used [147].

#### 4.7.2. Metabolic Labeling with Azide-Modified GlcNAc (GlcNAz)

Metabolic labeling with GlcNAz is a technique used to incorporate azide-modified GlcNAc into cellular proteins, allowing for the detection of O-GlcNAc modifications [148].

Cells are cultured in the presence of azide-modified GlcNAc (GlcNAz). The cells incorporate GlcNAz into proteins in place of the normal GlcNAc, resulting in the azide-modification of O-GlcNAc sites [149]. The azide-modified proteins are then subjected to a bioorthogonal reaction, such as click chemistry, to covalently attach a reporter tag (e.g., a fluorophore or biotin) to the azide group [150]. The tagged proteins can be enriched and purified using affinity methods if a biotin tag is used. The labeled proteins are detected and analyzed using techniques like Western blotting, fluorescence microscopy, or mass spectrometry, depending on the reporter tag and the experimental design [150].

### 4.8. Hydroxylation

#### 4.8.1. Proximity Ligation Assay (PLA)

The proximity ligation assay (PLA) is a sensitive method used to detect and visualize protein modifications, including hydroxylation, at the single-molecule level in cells [150]. Cells or tissue samples are fixed and permeabilized to allow access to the antibodies. Two primary antibodies are used: one that specifically recognizes the protein of interest and another that recognizes the hydroxylation modification [150]. Secondary antibodies, known as PLA probes, which are conjugated to oligonucleotides, are added. These probes bind to the primary antibodies [151]. If the PLA probes are in close proximity (due to binding to the target and modification on the same protein), a ligation reaction occurs, followed by an amplification step [151]. This amplification generates a fluorescent signal that can be detected using fluorescence microscopy. The fluorescent signals, each representing a single hydroxylated protein, are visualized and quantified using fluorescence microscopy, providing information on the localization and abundance of the hydroxylation modification in cells [152].

#### 4.8.2. Stable Isotope Labeling by Amino Acids in Cell Culture (SILAC) Combined with Mass Spectrometry

SILAC combined with mass spectrometry is a powerful technique for quantitative proteomics, including the study of hydroxylation [153]. Cells are grown in a medium containing stable isotope-labeled amino acids (e.g., heavy lysine and arginine) [154]. Proteins synthesized in these conditions incorporate these labeled amino acids. Proteins are extracted from the cells, and hydroxylated proteins or peptides are enriched using specific antibodies or chemical methods [154]. The enriched proteins are analyzed by mass spectrometry. The use of SILAC allows for quantitative comparison between samples, as the mass difference introduced by the heavy amino acids can be detected, indicating the extent of hydroxylation [153]. The mass spectrometry data is processed to quantify the hydroxylation levels and to identify specific hydroxylation sites on proteins [154].

### 4.9. Sulfenation

#### 4.9.1. Biotin Switch Technique

The biotin switch technique is a widely used method for detecting cysteine sulfenation in proteins [155].

Proteins are extracted from cells or tissues under non-reducing conditions to preserve sulfenic acid modifications [143]. Free cysteine thiols are blocked using a thiol-specific reagent, such as methyl methanethiosulfonate (MMTS), to prevent non-specific labeling. Sulfenic acids (SOH) are specifically reduced to free thiols, typically using a reducing agent like ascorbate [143]. The newly generated thiols are labeled with a biotinylated reagent, allowing for the selective detection of sulfenated cysteines [143]. Biotinylated proteins are enriched using streptavidin beads and then detected by Western blotting or mass spectrometry. This process allows for the identification of proteins that undergo sulfenation and the specific cysteine residues modified [152].

#### 4.9.2. Resin-Assisted Capture (RAC)

Resin-assisted capture (RAC) is a technique used for the enrichment and identification of cysteine-modified peptides, including sulfenation [151]. Proteins are extracted and then digested into peptides. Free cysteine residues are alkylated to prevent non-specific binding, typically using iodoacetamide or N-ethylmaleimide [151]. Cysteine-modified peptides are selectively captured on a thiol-reactive resin. The resin specifically binds to cysteine-containing peptides, including those with sulfenic acid modifications [156]. The captured peptides are released from the resin and can be labeled for further analysis, often with isotopic or isobaric tags for quantitative mass spectrometry. The labeled peptides are analyzed by mass spectrometry to identify sulfenated cysteine residues and quantify the extent of sulfenation [157].

## 5. Emerging Role of PTMS towards Therapeutics

Experimental evidence has demonstrated the existence of over 600 different PTMs [92]. This diverse array of PTMs intricately shapes the proteome, exerting control over both the structural and functional attributes of proteins. Given their pivotal role in regulating cellular processes and influencing the aging process, the precise control of protein PTMs becomes imperative. Dysregulation of PTMs has the potential to significantly contribute to the pathogenesis or progression of diseases [93,94].

Recent breakthroughs in MS and other cutting-edge analytical methodologies have significantly advanced our capabilities for pinpointing and comprehending PTMs within a wide spectrum of proteomes [158]. These investigations have brought to light the ubiquity and intricacy of PTMs, encompassing an array of modifications, including phosphorylation, acetylation, methylation, ubiquitination, SUMOylation, and glycosylation, to name a few [159]. Importantly, it is now well-established that PTMs exhibit dynamic regulation 1691 in response to a diverse range of stimuli, including hormonal cues [160], growth factors [161], environmental stressors [162], and microbial infections [163]. These revelations underscore the evolving landscape of PTM research and its profound implications for our understanding of cellular regulation and disease pathology.

Histone deacetylases (HDACs) that eliminate acetyl groups from histone and non-histone proteins have also been reported as playing an emerging role towards the treatment of neurodegenerative disorders and some inflammatory diseases [164,165,166]. Additionally, other PTM-targeting drugs, including proteasome inhibitors such as bortezomib for multiple myeloma and intense and treatment-resistant instances of anti-NMDA receptor (anti-NMDAR) encephalitis [167,168].

PTMs have a wide range of applications in the field of drug designing. The drug induced modifications can be used as PTM-targeted medications and also to verify the inhibition of pharmacologically important protein targets [169,170,171]. For example, drugs can be used to inhibit the multifunctional disordered protein and the measure of alterations in the PTM profile could be monitored as evidenced by PLA [172,173]. Additionally, following the isolation of SUMOylated proteins, Western blot can be used as a potential tool to demonstrate the modifications in the SUMOylated partner proteins of NUPR1 in response to various pharmacological treatments [174,175]. These examples highlight that PTMs can be used to design innovative therapeutics against critical protein targets and allow researchers to indirectly modulate the functions of protein of interest. This is a conventional and promising approach in drug design [175].

**Table 1:** Comparison table for the specified PTMs and their respective techniques.

PTM Technique	Analytical Method	Applications	Example	Limitations	Recent Developments	References
Methylation	Mass Spectrometry (MS)	Identifying and quantifying methylation sites; epigenetics and gene regulation studies. Comprehensive coverage of DNA methylation across the genome.	DNA methylation leads to progression of Alzheimer’s disease. [126,132] Hyper methylation of APP in Alzheimer’s disease [176].	Requires sophisticated equipment; complex data analysis. High cost; complexity in data analysis.	Improved MS sensitivity and resolution for low-abundance peptides. Advances in sequencing technologies for more efficient and cost-effective analysis.	[13,143]
Phosphorylation	Proximity Ligation Assay (PLA)	Detects phosphorylation in situ; used in signal transduction studies.	Phosphorylation of tau protein in Alzheimer’s disease [36,177].	Limited to proteins with available specific antibodies.	More sensitive probes and automated image analysis.	[16]
	Phosphoproteomics	Large-scale study of phosphorylated proteins; useful in disease research.	Phosphorylation of alpha-synuclein with the aid of kinases and phosphatases in PD [36].	Complex sample preparation; data interpretation challenges.	Improved enrichment techniques for phosphopeptides.	[131]
Sumoylation	SIMS (Sumoylation Identification by Mass Spectrometry)	Identifies sumoylation sites on proteins; useful in studying protein function and regulation.	Sumoylation of alpha-synuclein protein leads to progression of Parkinson’s disease [134,166].	Requires advanced mass spectrometry techniques; complex sample preparation.	Enhanced sensitivity and accuracy in sumoylation site identification.	[134,166]
	SuPrPA (SUMO Protease Protection Assay)	Studies SUMO conjugation/deconjugation dynamics.	Sumoylation of beta-secretases leads to increased amyloid-beta production leading to onset of Alzheimer’s disease [178].	Limited by the availability and specificity of SUMO proteases.	Development of more specific and sensitive assays for SUMO dynamics.	[166]
Ubiquitination	Ubiquitin Enrichment	Enriches ubiquitinated proteins; studies protein degradation and signaling pathways.	Ubiquitination of ligases result in neurodegenerative conditions [69,97].	Potential for non-specific binding; requires careful control.	Development of more specific enrichment strategies.	[144]
	TUBEs (Tandem Ubiquitin Binding Entities)	Enriches ubiquitinated proteins; studies ubiquitin-mediated processes.	PARK2 is a ubiquitin-signaling gene that is associated with PD [179].	Potential for non-specific binding; requires careful control.	Development of more specific TUBEs for different ubiquitin chains.	[135]
Nitration	Immunoprecipitation combined with Mass Spectrometry (IP-MS)	Detects and quantifies protein nitration; used in oxidative stress studies.	Tyrosine nitration is an earliest marker of Alzheimer’s disease. [139,141]	Specificity depends on antibody quality; complex sample preparation.	Improved antibody specificity and mass spectrometry techniques.	[141]
	LC-MS/MS (Liquid Chromatography-Mass Spectrometry/Mass Spectrometry)	Quantitative analysis of protein nitration; studies nitrosative stress.	Nitration of alpha-synuclein leads to increased aggregation and PD pathogenesis [180].	Complex sample preparation; requires high-resolution mass spectrometry.	Advances in LC-MS/MS for improved detection and quantification.	[138,139]
Truncation	N-Terminal COFRADIC	Analyzes protein N-termini; identifies truncation events.	Truncation of Tau protein in Alzheimer’s disease. [143,144]	Technically challenging; requires specialized equipment.	Improved methods for N-terminal peptide enrichment and analysis.	[144]
	C-Terminal Sequencing	Identifies protein C-terminal truncations; useful in studying proteolysis.	Truncation of alpha-synuclein and amyloid-beta leads to neurodegenerative conditions [181]	Limited availability of techniques for C-terminal analysis.	Development of more efficient C-terminal sequencing methods.	[143,181]
O-GlcNAcylation	Chemoenzymatic Labeling	Detects O-GlcNAc modifications; studies cellular signaling and regulation.	Dysregulated O-GlcNAcylation of Alpha-synuclein leads to Parkinsonism. [82,90,94]	Requires specific enzymes and bioorthogonal chemistry.	Development of more efficient labeling and detection methods.	[149]
	GlcNAz (Metabolic Labeling with Azide-Modified GlcNAc)	Incorporates azide-modified GlcNAc into proteins; studies O-GlcNAcylation dynamics.	O-GlcNAcylation of amyloid-beta have neuroprotective effects in Alzheimer’s disease [182].	Requires cell permeable and metabolically incorporated azide-GlcNAc.	Improved azide-GlcNAc analogs for better incorporation and detection.	[81]
Hydroxylation	Proximity Ligation Assay (PLA)	Detects hydroxylation in situ; used in hypoxia and collagen biosynthesis studies.	Proline Hydroxylation in the brain leads to activation of kinases leading to inflammation. [38,42]	Limited to proteins with available specific antibodies.	More sensitive probes and automated image analysis.	[155]
	SILAC combined with Mass Spectrometry	Quantitative analysis of hydroxylation; studies protein stability and signaling.	Tyrosine hydroxylation leads to PD pathogenesis [183].	Requires metabolic labeling; complex sample preparation.	Advances in SILAC and MS for improved quantification and sensitivity.	[154]
Sulfenation	Biotin Switch Technique	Detects cysteine sulfenation; studies redox regulation and signaling.	Sulfenation of cysteine residues leads to onset of Parkinsonism. [156,157]	Potential for non-specific labeling; requires careful control.	Development of more specific reagents for sulfenic acid detection.	[156]
	Resin-Assisted Capture (RAC)	Enriches cysteine-modified peptides; identifies sulfenation sites.	Sulfation of tau, Aβ, and α-synuclein aggregates aided by heparan sulfate proteoglycans (HSPGs) leads to neurodegenerative conditions [184].	Requires specific capture resins; complex sample preparation.	Improved resin materials for more efficient capture and release.	[151,184]

In summary, PTMs are essential regulators of protein function and have potentially significant implications for understanding and treating human diseases. Developing effective PTM-targeting therapeutics requires a comprehensive understanding of the specific PTM involved, its regulatory enzymes or factors, and its downstream effects on cellular processes. Therefore, continued research in this field is crucial for advancing precision medicine and improving patient outcomes.

## 6. Conclusions

Post-translational modifications (PTMs) exert a profound influence on diverse facets of cellular biology and offer invaluable perspectives into the intricate machinery of biological and pathological processes. They significantly augment the intricacy of living organisms. In the pursuit of developing efficacious therapeutics targeting PTMs, a holistic grasp of the given PTM, its intertwined regulatory elements and enzymatic players, as well as its downstream consequences on cellular functions is indispensable. Recognizing this significance, persistent investigations in this realm stand as a critical imperative, holding the potential to propel the frontiers of precision medicine and ameliorate patient prognosis.

## References

[1] Chen L., Kashina A. (2021). Post-translational Modifications of the Protein Termini. Front. Cell Dev. Biol..

[2] Ramazi S., Zahiri J. (2021). Post-translational modifications in proteins: Resources, tools and prediction methods. Database.

[3] Mann M., Jensen O.N. (2003). Proteomic analysis of post-translational modifications. Nat. Biotechnol..

[4] Waddell J., Banerjee A., Kristian T. (2021). Acetylation in Mitochondria Dynamics and Neurodegeneration. Cells.

[5] Shashi V., Magiera M.M., Klein D., Zaki M., Schoch K., Rudnik-Schöneborn S., Norman A., Lopes Abath Neto O., Dusl M., Yuan X. (2018). Loss of tubulin deglutamylase CCP1 causes infantile-onset neurodegeneration. EMBO J..

[6] Bowie J.U., Post C.B. (2015). The 29th Annual Symposium of the Protein Society, Barcelona, Spain, July 22–25. Protein Sci..

[7] Duan G., Walther D. (2015). The Roles of Post-translational modifications in the context of protein interaction networks. PLoS Comput. Biol..

[8] Liu Y.-P., Zhang T.-N., Wen R., Liu C.-F., Yang N. (2022). Role of Posttranslational Modifications of Proteins in Cardiovascular Disease. Oxidative Med. Cell. Longev..

[9] Zhong Q., Xiao X., Qiu Y., Xu Z., Chen C., Chong B., Zhao X., Hai S., Li S., An Z. (2023). Protein posttranslational modifications in health and diseases: Functions, regulatory mechanisms, and therapeutic implications. Medcomm.

[10] Yang Y.-H., Wen R., Yang N., Zhang T.-N., Liu C.-F. (2023). Roles of protein post-translational modifications in glucose and lipid metabolism: Mechanisms and perspectives. Mol. Med..

[11] Gajjala P.R., Fliser D., Speer T., Jankowski V., Jankowski J. (2015). Emerging role of post-translational modifications in chronic kidney disease and cardiovascular disease. Nephrol. Dial. Transplant..

[12] Gupta R., Sahu M., Srivastava D., Tiwari S., Ambasta R.K., Kumar P. (2021). Post-translational modifications: Regulators of neurodegenerative proteinopathies. Ageing Res. Rev..

[13] Huang K.-Y., Lee T.-Y., Kao H.-J., Ma C.-T., Lee C.-C., Lin T.-H., Chang W.-C., Huang H.-D. (2018). dbPTM in 2019: Exploring disease association and cross-talk of post-translational modifications. Nucleic Acids Res..

[14] Walsh G., Jefferis R. (2006). Post-translational modifications in the context of therapeutic proteins. Nat. Biotechnol..

[15] Ong S.-E., Mann M.A. (2006). practical recipe for stable isotope labeling by amino acids in cell culture (SILAC). Nat. Protoc..

[16] Hunter T. (2000). Signaling—And Beyond. Cell.

[17] Röhl A., Rohrberg J., Buchner J. (2013). The chaperone Hsp90: Changing partners for demanding clients. Trends Biochem. Sci..

[18] Sahasrabudhe P., Rohrberg J., Biebl M.M., Rutz D.A., Buchner J. (2017). The Plasticity of the Hsp90 Co-chaperone System. Mol. Cell.

[19] Cox M.B., Johnson J.L. (2018). Evidence for Hsp90 Co-chaperones in Regulating Hsp90 Function and Promoting Client Protein Folding. Chaperones: Methods and Protocols.

[20] Rakhit R., Chakrabartty A. (2006). Structure, folding, and misfolding of Cu, Zn superoxide dismutase in amyotrophic lateral sclerosis. Biochim. Biophys. Acta (BBA)-Mol. Basis Dis..

[21] Arai T., Hasegawa M., Akiyama H., Ikeda K., Nonaka T., Mori H., Mann D., Tsuchiya K., Yoshida M., Hashizume Y. (2006). TDP-43 is a component of ubiquitin-positive tau-negative inclusions in frontotemporal lobar degeneration and amyotrophic lateral sclerosis. Biochem. Biophys. Res. Commun..

[22] Withers J., Dong X. (2017). Post-translational regulation of plant immunity. Curr. Opin. Plant Biol..

[23] Malik N., Ferreira B.I., Hollstein P.E., Curtis S.D., Trefts E., Weiser Novak S., Yu J., Gilson R., Hellberg K., Fang L. (2023). Induction of lysosomal and mitochondrial biogenesis by AMPK phosphorylation of FNIP1. Science.

[24] Gavin A.-C., Bösche M., Krause R., Grandi P., Marzioch M., Bauer A., Schultz J., Rick J.M., Michon A.-M., Cruciat C.-M. (2002). Functional organization of the yeast proteome by systematic analysis of protein complexes. Nature.

[25] Shubchynskyy V., Boniecka J., Schweighofer A., Simulis J., Kvederaviciute K., Stumpe M., Mauch F., Balazadeh S., Mueller-Roeber B., Boutrot F. (2017). Protein phosphatase AP2C1 negatively regulates basal resistance and defense responses to Pseudomonas syringae. J. Exp. Bot. Erw..

[26] Ji F., Zhou M., Zhu H., Jiang Z., Li Q., Ouyang X., Lv Y., Zhang S., Wu T., Li L. (2022). Integrative Proteomic Analysis of Multiple Posttranslational Modifications in Inflammatory Response. Genom. Proteom. Bioinform..

[27] Aillaud C., Bosc C., Peris L., Bosson A., Heemeryck P., Van Dijk J., Le Friec J., Boulan B., Vossier F., Sanman L.E. (2017). Vaso-hibins/SVBP are tubulin carboxypeptidases (TCPs) that regulate neuron differentiation. Science.

[28] Brazil D.P., Hemmings B.A. (2001). Ten years of protein kinase B signalling: A hard Akt to follow. Trends Biochem. Sci..

[29] Persad S., Attwell S., Gray V., Mawji N., Deng J.T., Leung D., Yan J., Sanghera J., Walsh M.P., Dedhar S. (2001). Regulation of Protein Kinase B/Akt-Serine 473 Phosphorylation by Integrin-linked Kinase. J. Biol. Chem..

[30] Patel M., Sachidanandan M., Adnan M. (2019). Serine arginine protein kinase 1 (SRPK1): A moonlighting protein with theranostic ability in cancer prevention. Mol. Biol. Rep..

[31] Kenwood B.M., Weaver J.L., Bajwa A., Poon I.K., Byrne F.L., Murrow B.A., Calderone J.A., Huang L., Divakaruni A.S., Tomsig J.L. (2014). Identification of a novel mitochondrial uncoupler that does not depolarize the plasma membrane. Mol. Metab..

[32] Liu Y.-Z., Chrivia J.C., Latchman D.S. (1998). Nerve Growth Factor Up-regulates the Transcriptional Activity of CBP through Ac-tivation of the p42/p44MAPK Cascade. J. Biol. Chem..

[33] Alberini C.M. (2009). Transcription Factors in Long-Term Memory and Synaptic Plasticity. Physiol. Rev..

[34] Chrivia J.C., Kwok R.P.S., Lamb N., Hagiwara M., Montminy M.R., Goodman R.H. (1993). Phosphorylated CREB binds specifically to the nuclear protein CBP. Nature.

[35] Illenberger S., Zheng-Fischhöfer Q., Preuss U., Stamer K., Baumann K., Trinczek B., Biernat J., Godemann R., Mandelkow E.M., Mandelkow E. (1998). The endogenous and cell cycle-dependent phosphorylation of tau protein in living cells: Implications for Alzheimer’s disease. Mol. Biol. Cell.

[36] Noble W., Hanger D.P., Miller C.C.J., Lovestone S. (2013). The Importance of Tau Phosphorylation for Neurodegenerative Diseases. Front. Neurol..

[37] Kaelin W.G., Ratcliffe P.J. (2008). Oxygen sensing by metazoans: The central role of the HIF hydroxylase pathway. Mol. Cell.

[38] Hutton J.J., Kaplan A., Udenfriend S. (1967). Conversion of the amino acid sequence gly-pro-pro in protein to gly-pro-hyp by collagen proline hydroxylase. Arch. Biochem. Biophys..

[39] Hausinger R.P. (2004). Fe(II)/α-Ketoglutarate-Dependent Hydroxylases and Related Enzymes. Crit. Rev. Biochem. Mol. Biol..

[40] Rappu P., Salo A.M., Myllyharju J., Heino J. (2019). Role of prolyl hydroxylation in the molecular interactions of collagens. Essays Biochem..

[41] Salo A.M., Myllyharju J. (2021). Prolyl and lysyl hydroxylases in collagen synthesis. Exp. Dermatol..

[42] Kirchner M., Deng H., Xu Y. (2021). Heterogeneity in proline hydroxylation of fibrillar collagens observed by mass spectrometry. PLoS ONE.

[43] Ferrante F., Giaimo B.D., Friedrich T., Sugino T., Mertens D., Kugler S., Gahr B.M., Just S., Pan L., Bartkuhn M. (2022). Hydroxylation of the NOTCH1 intracellular domain regulates Notch signaling dynamics. Cell Death Dis..

[44] Cai Z., Zhao B., Deng Y., Shangguan S., Zhou F., Zhou W., Li X., Li Y., Chen G. (2016). Notch signaling in cerebrovascular diseases (Review). Mol. Med. Rep..

[45] Jakovljevic A., Nikolic N., Paternò Holtzman L., Tournier P., Gaudin A., Cordaro L., Milinkovic I. (2023). Involvement of the Notch signaling system in alveolar bone resorption. Jpn. Dent. Sci. Rev..

[46] Lee D.Y., Teyssier C., Strahl B.D., Stallcup M.R. (2005). Role of Protein Methylation in Regulation of Transcription. Endocr. Rev..

[47] Kouzarides T. (2007). Chromatin Modifications and Their Function. Cell.

[48] Shi Y., Lan F., Matson C., Mulligan P., Whetstine J.R., Cole P.A., Casero R.A., Shi Y. (2004). Histone Demethylation Mediated by the Nuclear Amine Oxidase Homolog LSD1. Cell.

[49] Lee Y.-H., Stallcup M.R. (2009). Minireview: Protein Arginine Methylation of Nonhistone Proteins in Transcriptional Regulation. Mol. Endocrinol..

[50] Kim J., Daniel J., Espejo A., Lake A., Krishna M., Xia L., Zhang Y., Bedford M.T. (2006). Tudor, MBT and chromo domains gauge the degree of lysine methylation. Embo. Rep..

[51] Scoumanne A., Zhang J., Chen X. (2009). PRMT5 is required for cell-cycle progression and p53 tumor suppressor function. Nucleic Acids Res..

[52] Carr S.M., Munro S., Sagum C.A., Fedorov O., Bedford M.T., La Thangue N.B. (2017). Tudor-domain protein PHF20L1 reads lysine methylated retinoblastoma tumour suppressor protein. Cell Death Differ..

[53] Huang J., Sengupta R., Espejo A.B., Lee M.G., Dorsey J.A., Richter M., Opravil S., Shiekhattar R., Bedford M.T., Jenuwein T. (2007). p53 is regulated by the lysine demethylase LSD1. Nature.

[54] Balmik A.A., Chinnathambi S. (2021). Methylation as a key regulator of Tau aggregation and neuronal health in Alzheimer’s disease. Cell Commun. Signal..

[55] Sommerer Y., Dobricic V., Schilling M., Ohlei O., Sabet S.S., Wesse T., Fuß J., Franzenburg S., Franke A., Parkkinen L. (2023). Entorhinal cortex epigenome-wide association study highlights four novel loci showing differential methylation in Alzheimer’s disease. Alzheimers Res. Ther..

[56] Sacksteder C.A., Qian W.-J., Knyushko T.V., Wang H., Chin M.H., Lacan G., Melega W.P., Camp D.G., Smith R.D., Smith D.J. (2006). Endogenously Nitrated Proteins in Mouse Brain: Links to Neurodegenerative Disease. Biochemistry.

[57] Beckman J.S., Beckman T.W., Chen J., Marshall P.A., Freeman B.A. (1990). Apparent hydroxyl radical production by peroxynitrite: Implications for endothelial injury from nitric oxide and superoxide. Proc. Natl. Acad. Sci.USA.

[58] Torreilles F., Salman-Tabcheh S., Guérin M.-C., Torreilles J. (1999). Neurodegenerative disorders: The role of peroxynitrite. Brain Res. Rev..

[59] Manzanza N.D.O., Sedlackova L., Kalaria R.N. (2021). Alpha-Synuclein Post-translational Modifications: Implications for Patho-genesis of Lewy Body Disorders. Front. Aging Neurosci..

[60] Amagai R., Yoshioka S., Otomo R., Nagano H., Hashimoto N., Sakakibara R., Tanaka T., Okado-Matsumoto A. (2023). Post-translational modification of lysine residues in erythrocyte α-synuclein. J. Biochem..

[61] Takahashi T., Yamashita H., Nakamura T., Nagano Y., Nakamura S. (2002). Tyrosine 125 of α-synuclein plays a critical role for dimerization following nitrative stress. Brain Res..

[62] Gómez-Tortosa E., Gonzalo I., Newell K., Yébenes J., Vonsattel J., Hyman B. (2002). Patterns of protein nitration in dementia with Lewy bodies and striatonigral degeneration. Acta Neuropathol..

[63] Reynolds M.R., Berry R.W., Binder L.I. (2005). Site-Specific Nitration and Oxidative Dityrosine Bridging of the τ Protein by Perox-ynitrite: Implications for Alzheimer’s Disease. Biochemistry.

[64] Horiguchi T., Uryu K., Giasson B.I., Ischiropoulos H., LightFoot R., Bellmann C., Richter-Landsberg C., Lee V.M.-Y., Trojanowski J.Q. (2003). Nitration of Tau Protein Is Linked to Neurodegeneration in Tauopathies. Am. J. Pathol..

[65] Haines J.D., Inglese M., Casaccia P. (2011). Axonal Damage in Multiple Sclerosis. Mt. Sinai J. Med. A J. Transl. Pers. Med..

[66] Kean R.B., Spitsin S.V., Mikheeva T., Scott G.S., Hooper D.C. (2000). The Peroxynitrite Scavenger Uric Acid Prevents Inflammatory Cell Invasion into the Central Nervous System in Experimental Allergic Encephalomyelitis through Maintenance of Blood-Central Nervous System Barrier Integrity. J. Immunol..

[67] Roos G., Messens J. (2011). Protein sulfenic acid formation: From cellular damage to redox regulation. Free Radic. Biol. Med..

[68] Reddie K.G., Carroll K.S. (2008). Expanding the functional diversity of proteins through cysteine oxidation. Curr. Opin. Chem. Biol..

[69] Gupta V., Carroll K.S. (2014). Sulfenic acid chemistry, detection and cellular lifetime. Biochim. Biophys. Acta (BBA)-General. Subj..

[70] Meng F., Yao D., Shi Y., Kabakoff J., Wu W., Reicher J., Ma Y., Moosmann B., Masliah E., Lipton S.A. (2011). Oxidation of the cysteine-rich regions of parkin perturbs its E3 ligase activity and contributes to protein aggregation. Mol. Neurodegener..

[71] Nakamura T., Tu S., Akhtar M.W., Sunico C.R., Okamoto S.I., Lipton S.A. (2013). Review Aberrant Protein S-Nitrosylation in Neuro-degenerative Diseases. Neuron.

[72] Karplus P.A. (2015). A Primer on Peroxiredoxin Biochemistry. Free Radic. Biol. Med..

[73] Karplus P.A., Poole L.B. (2012). Peroxiredoxins as molecular triage agents, sacrificing themselves to enhance cell survival during a peroxide attack. Mol. Cell.

[74] Xiong A., Yang Z., Shen Y., Zhou J., Shen Q. (2014). Transcription Factor STAT3 as a Novel Molecular Target for Cancer Prevention. Cancers.

[75] Sobotta M.C., Liou W., Stöcker S., Talwar D., Oehler M., Ruppert T., Scharf A.N.D., Dick T.P. (2014). Peroxiredoxin-2 and STAT3 form a redox relay for H_2_O_2_ signaling. Nat. Chem. Biol..

[76] Lee S.R., Yang K.S., Kwon J., Lee C., Jeong W., Rhee S.G. (2002). Reversible inactivation of the tumor suppressor PTEN by H_2_O_2_. J. Biol. Chem..

[77] Awata H., Huang C., Handlogten M.E., Miller R.T. (2001). Interaction of the Calcium-sensing Receptor and Filamin, a Potential Scaffolding Protein. J. Biol. Chem..

[78] Paulsen C.E., Truong T.H., Garcia F.J., Homann A., Gupta V., Leonard S.E., Carroll K.S. (2011). Peroxide-dependent sulfenylation of the EGFR catalytic site enhances kinase activity. Nat. Chem. Biol..

[79] Paulsen C.E., Carroll K.S. (2013). Cysteine-Mediated Redox Signaling: Chemistry, Biology, and Tools for Discovery. Chem. Rev..

[80] Scheibe F., Prüss H., Mengel A.M., Kohler S., Nümann A., Köhnlein M., Ruprecht K., Alexander T., Hiepe F., Meisel A. (2017). Bortezomib for treatment of therapy-refractory anti-NMDA receptor encephalitis. Neurology.

[81] Hart G.W., Housley M.P., Slawson C. (2007). Cycling of O-linked beta-N-acetylglucosamine on nucleocytoplasmic proteins. Nature.

[82] Ma J., Li Y., Hou C., Wu C. (2021). O-GlcNAcAtlas: A database of experimentally identified O-GlcNAc sites and proteins. Glycobiology.

[83] Wells L., Vosseller K., Cole R.N., Cronshaw J.M., Matunis M.J., Hart G.W. (2002). Mapping Sites of O-GlcNAc Modification Using Affinity Tags for Serine and Threonine Post-translational Modifications. Mol. Cell. Proteom..

[84] Yang W.H., Kim J.E., Nam H.W., Ju J.W., Kim H.S., Kim Y.S., Cho J.W. (2006). Modification of p53 with O-linked N-acetylglucosamine regulates p53 activity and stability. Nat. Cell Biol..

[85] Hart G.W. (1997). Dynamic o-linked glycosylation of nuclear and cytoskeletal proteins. Annu. Rev. Biochem..

[86] Wells L., Gao Y., Mahoney J.A., Vosseller K., Rosen A., Hart G.W. (2002). Dynamic O-Glycosylation of Nuclear and Cytosolic Proteins: Further characterization of the nucleocytoplasmic β-N-acetylglucosaminidase, O-GlcNAcase. J. Biol. Chem..

[87] Bond M.R., Hanover J.A. (2013). O-GlcNAc Cycling: A Link Between Metabolism and Chronic Disease. Annu. Rev. Nutr..

[88] Yang X., Ongusaha P.P., Miles P.D., Havstad J.C., Zhang F., So W.V., Kudlow J.E., Michell R.H., Olefsky J.M., Field S.J. (2008). Phosphoinositide signalling links O-GlcNAc transferase to insulin resistance. Nature.

[89] Yang X., Qian K. (2017). Protein O-GlcNAcylation: Emerging mechanisms and functions. Nat. Rev. Mol. Cell Biol..

[90] Toleman C.A., Schumacher M.A., Yu S.-H., Zeng W., Cox N.J., Smith T.J., Soderblom E.J., Wands A.M., Kohler J.J., Boyce M. (2018). Structural basis of O-GlcNAc recognition by mammalian 14-3-3 proteins. Proc. Natl. Acad. Sci. USA.

[91] Zhu Y., Liu T.-W., Madden Z., Yuzwa S.A., Murray K., Cecioni S., Zachara N., Vocadlo D.J. (2016). Post-translational O-GlcNAcylation is essential for nuclear pore integrity and maintenance of the pore selectivity filter. J. Mol. Cell Biol..

[92] Zachara N.E., O’Donnell N., Cheung W.D., Mercer J.J., Marth J.D., Hart G.W. (2004). Dynamic O-GlcNAc Modification of Nucleo-cytoplasmic Proteins in Response to Stress A Survival Response Of Mammalian Cells. J. Biol. Chem..

[93] Zou L., Yang S., Hu S., Chaudry I.H., Marchase R.B., Chatham J.C. (2007). The protective effects of PUGNAc on cardiac function after trauma-hemorrhage are mediated via increased protein O-GlcNAc levels. Shock.

[94] Lee A., Miller D., Henry R., Paruchuri V.D.P., O’Meally R.N., Boronina T., Cole R.N., Zachara N.E. (2016). Combined Antibody/Lectin Enrichment Identifies Extensive Changes in the O-GlcNAc Sub-proteome upon Oxidative Stress. J. Proteome Res..

[95] Zafar S., Shafiq M., Younas N., Schmitz M., Ferrer I., Zerr I. (2017). Prion Protein Interactome: Identifying Novel Targets in Slowly and Rapidly Progressive Forms of Alzheimer’s Disease. J. Alzheimers Dis..

[96] Pickart C.M. (2001). Mechanisms Underlying Ubiquitination. Annu. Rev. Biochem..

[97] Komander D., Rape M. (2012). The ubiquitin code. Annu. Rev. Biochem..

[98] Reyes-Turcu F.E., Horton J.R., Mullally J.E., Heroux A., Cheng X., Wilkinson K.D. (2006). The Ubiquitin Binding Domain ZnF UBP Recognizes the C-Terminal Diglycine Motif of Unanchored Ubiquitin. Cell.

[99] Meluh P.B., Koshland D. (1995). Evidence that the MIF2 gene of Saccharomyces cerevisiae encodes a centromere protein with homology to the mammalian centromere protein CENP-C. Mol. Biol. Cell.

[100] Ikeda F., Dikic I. (2008). Atypical ubiquitin chains: New molecular signals. ‘Protein Modifications: Beyond the Usual Suspects’ Review Series. EMBO Rep..

[101] Flotho A., Melchior F. (2013). Sumoylation: A regulatory protein modification in health and disease. Annu. Rev. Biochem..

[102] Pichler A., Fatouros C., Lee H., Eisenhardt N. (2017). SUMO conjugation—A mechanistic view. Biomol. Concepts.

[103] Soares E.S., Prediger R.D., Brocardo P.S., Cimarosti H.I. (2022). SUMO-modifying Huntington’s disease. IBRO Neurosci. Rep..

[104] Hannoun Z., Fletcher J., Greenhough S., Medine C., Samuel K., Sharma R., Pryde A., Black J.R., Ross J.A., Wilmut I. (2010). The comparison between conditioned media and serum-free media in human embryonic stem cell culture and differentiation. Cell Reprogram.

[105] Mukhopadhyay D., Dasso M. (2007). Modification in reverse: The SUMO proteases. Trends Biochem. Sci..

[106] Hay R.T. (2004). Modifiying NEMO. Nat. Cell Biol..

[107] Kuhn P.H., Koroniak K., Hogl S., Colombo A., Zeitschel U., Willem M., Volbracht C., Schepers U., Imhof A., Hoffmeister A. (2012). Secretome protein enrichment identifies physiological BACE1 protease substrates in neurons. EMBO J..

[108] Rogers L.D., Overall C.M. (2013). Proteolytic Post-translational Modification of Proteins: Proteomic Tools and Methodology. Mol. Cell. Proteom..

[109] Muzio M., Stockwell B.R., Stennicke H.R., Salvesen G.S., Dixit V.M. (1998). An Induced Proximity Model for Caspase-8 Activation. J. Biol. Chem..

[110] Stennicke H.R., Jürgensmeier J.M., Shin H., Deveraux Q., Wolf B.B., Yang X., Zhou Q., Ellerby H.M., Ellerby L.M., Bredesen D. (1998). Pro-caspase-3 is a major physiologic target of Caspase-8. J. Biol. Chem..

[111] Rohn T.T., Head E., Nesse W.H., Cotman C.W., Cribbs D.H. (2001). Activation of Caspase-8 in the Alzheimer’s Disease Brain. Neurobiol. Dis..

[112] Mikhailova A.G., Nekrasov A.N., Zinchenko A.A., Rakitina T.V., Korzhenevsky D.A., Lipkin A.V., Razguljaeva O.A., Ovchinnikova M.V., Gorlenko V.A., Rumsh L.D. (2015). Truncated Variants of Serratia proteamaculans Oligopeptidase B Having Different Activities. Biochemistry.

[113] Fortini M.E. (2009). Notch signaling: The core pathway and its posttranslational regulation. Dev. Cell.

[114] Conover C.A., Oxvig C. (2017). PAPP-A: A promising therapeutic target for healthy longevity. Aging Cell.

[115] Oxvig C., Conover C.A. (2023). The Stanniocalcin-PAPP-A-IGFBP-IGF Axis. J. Clin. Endocrinol. Metab..

[116] Voutsadakis I.A. (2021). Mutations of p53 associated with pancreatic cancer and therapeutic implications. Ann. Hepatobiliary Pancreat Surg..

[117] Zhang Y.J., Xu Y.F., Cook C., Gendron T.F., Roettges P., Link C.D., Lin W.L., Tong J., Castanedes-Casey M., Ash P. (2009). Aberrant cleavage of TDP-43 enhances aggregation and cellular toxicity. Proc. Natl. Acad. Sci. USA.

[118] Johnson B.A., Murray E.D., Clarke S., Glass D.B., Aswad D.W. (1987). Protein carboxyl methyltransferase facilitates conversion of atypical L-isoaspartyl peptides to normal L-aspartyl peptides. J. Biol. Chem..

[119] Chatterjee T., Das G., Chatterjee B.K., Dhar J., Ghosh S., Chakrabarti P. (2020). The role of isoaspartate in fibrillation and its prevention by Protein-L-isoaspartyl methyltransferase. Biochim. Biophys. Acta (BBA)-General. Subj..

[120] Yang H., Lyutvinskiy Y., Herukka S.K., Soininen H., Rutishauser D., Zubarev R.A. (2014). Prognostic polypeptide blood plasma biomarkers of Alzheimer’s disease progression. J. Alzheimers Dis..

[121] Yang H., Wittnam J.L., Zubarev R.A., Bayer T.A. (2013). Shotgun brain proteomics reveals early molecular signature in presymp-tomatic mouse model of Alzheimer’s disease. J. Alzheimers Dis..

[122] Yang H., Lyutvinskiy Y., Soininen H., Zubarev R.A. (2011). Alzheimer’s disease and mild cognitive impairment are associated with elevated levels of isoaspartyl residues in blood plasma proteins. J. Alzheimers Dis..

[123] Fukuda H., Shimizu T., Nakajima M., Mori H., Shirasawa T. (1999). Synthesis, aggregation, and neurotoxicity of the Alzheimer’s Aβ1-42 amyloid peptide and its isoaspartyl isomers. Bioorg Med. Chem. Lett..

[124] Johnson B.A., Shirokawa J.M., Geddes J.W., Choi B.H., Kim R.C., Aswad D.W. (1991). Protein L-isoaspartyl methyltransferase in postmortem brains of aged humans. Neurobiol. Aging.

[125] Wang J., Zhang Y.R., Shen X.N., Han J., Cui M., Tan L., Dong Q., Zubarev R.A., Yu J.T. (2022). Deamidation-related blood biomarkers show promise for early diagnostics of neurodegeneration. Biomark. Res..

[126] Patterson K., Molloy L., Qu W., Clark S. (2011). DNA Methylation: Bisulphite Modification and Analysis. J. Vis. Exp..

[127] Aebersold R., Mann M. (2016). Mass-spectrometric exploration of proteome structure and function. Nature.

[128] Loo J.A. (2000). Electrospray ionization mass spectrometry: A technology for studying noncovalent macromolecular complexes. Int. J. Mass. Spectrom..

[129] Gillette M.A., Carr S.A. (2013). Quantitative analysis of peptides and proteins in biomedicine by targeted mass spectrometry. Nat. Methods.

[130] Hendriks I.A., Lyon D., Young C., Jensen L.J., Vertegaal A.C.O., Nielsen M.L. (2017). Site-specific mapping of the human SUMO proteome reveals co-modification with phosphorylation. Nat. Struct. Mol. Biol..

[131] Humphrey S.J., Yang G., Yang P., Fazakerley D.J., Stöckli J., Yang J.Y., James D.E. (2013). Dynamic adipocyte phosphoproteome reveals that akt directly regulates mTORC2. Cell Metab..

[132] Olova N., Krueger F., Andrews S., Oxley D., Berrens R.V., Branco M.R., Reik W. (2018). Correction to: Comparison of whole-genome bisulfite sequencing library preparation strategies identifies sources of biases affecting DNA methylation data. Genome Biol..

[133] Hendriks I.A., D’souza R.C., Yang B., Verlaan-De Vries M., Mann M., Vertegaal A.C. (2014). Uncovering global SUMOylation signaling networks in a site-specific manner. Nat. Struct. Mol. Biol..

[134] Li Y., Sun M., Hu Y., Shan Y., Liang Z., Zhang L., Zhang Y. (2021). Antibody-free enrichment method for proteome-wide analysis of endogenous SUMOylation sites. Anal. Chim. Acta.

[135] Hjerpe R., Aillet F., Lopitz-Otsoa F., Lang V., England P., Rodriguez M.S. (2009). Efficient protection and isolation of ubiquitylated proteins using tandem ubiquitin-binding entities. Embo. Rep..

[136] Kim W., Bennett E.J., Huttlin E.L., Guo A., Li J., Possemato A., Sowa M.E., Rad R., Rush J., Comb M.J. (2011). Systematic and Quantitative As-sessment of the Ubiquitin-Modified Proteome. Mol. Cell..

[137] Damgaard R.B. (2021). The ubiquitin system: From cell signalling to disease biology and new therapeutic opportunities. Cell Death Differ..

[138] Tyther R., McDonagh B., Sheehan D. (2011). Proteomics in investigation of protein nitration in kidney disease: Technical challenges and perspectives from the spontaneously hypertensive rat. Mass. Spectrom. Rev..

[139] Radi R. (2013). Protein tyrosine nitration: Biochemical mechanisms and structural basis of functional effects. Acc. Chem. Res..

[140] Kansanen E., Bonacci G., Schopfer F.J., Kuosmanen S.M., Tong K.I., Leinonen H., Woodcock S.R., Yamamoto M., Carlberg C., Ylä-Herttuala S. (2011). Electrophilic nitro-fatty acids activate NRF2 by a KEAP1 cysteine 151-independent mechanism. J. Biol. Chem..

[141] Feeney M.B., Schöneich C. (2013). Proteomic approaches to analyze protein tyrosine nitration. Antioxid. Redox Signal..

[142] Medeiros R., Sousa B., Rossi S., Afonso C., Bonino L., Pitt A., López E., Spickett C., Borthagaray G. (2021). Identification and relative quantification of 3-nitrotyrosine residues in fibrinogen nitrated in vitro and fibrinogen from ischemic stroke patient plasma using LC-MS/M.S. Free. Radic. Biol. Med..

[143] Ong S. (2003). Mass spectrometric-based approaches in quantitative proteomics. Methods.

[144] Stes E., Laga M., Walton A., Samyn N., Timmerman E., De Smet I., Goormachtig S., Gevaert K. (2014). A COFRADIC Protocol To Study Protein Ubiquitination. J. Proteome Res..

[145] Ma B., Khan K.S., Xu T., Amada J.X., Guo Z., Yan Y., Cheng A.S.L., Ng B.W.L. (2023). Targeted Protein O-GlcNAcylation Using Bifunctional Small Molecules. bioRxiv.

[146] Wu Z.L., Tatge T.J., Grill A.E., Zou Y. (2018). Detecting and Imaging O-GlcNAc Sites Using Glycosyltransferases: A Systematic Approach to Study O-GlcNAc. Cell Chem. Biol..

[147] Gorelik A., van Aalten D.M.F. (2020). Tools for functional dissection of site-specific O-GlcNAcylation. RSC Chem. Biol..

[148] Yoo T.Y., Mitchison T.J. (2021). *O*-GlcNAc modification of nuclear pore complexes accelerates bidirectional transport. J. Cell Biol..

[149] Zhang H., Qi J., Pei J., Zhang M., Shang Y., Li Z., Wang Y., Guo J., Sun K., Fan J. (2022). O-GlcNAc modification mediates aquaporin 3 to coordinate endometrial cell glycolysis and affects embryo implantation. J. Adv. Res..

[150] Söderberg O., Gullberg M., Jarvius M., Ridderstråle K., Leuchowius K.-J., Jarvius J., Wester K., Hydbring P., Bahram F., Larsson L.-G. (2006). Direct observation of individual endogenous protein complexes in situ by proximity ligation. Nat. Methods.

[151] Darmanis S., Nong R.Y., Vänelid J., Siegbahn A., Ericsson O., Fredriksson S., Bäcklin C., Gut M., Heath S., Gut I.G. (2011). ProteinSeq: High-performance proteomic analyses by proximity ligation and next generation sequencing. PLoS ONE.

[152] Cubeñas-Potts C., Srikumar T., Lee C., Osula O., Subramonian D., Zhang X.-D., Cotter R.J., Raught B., Matunis M.J. (2015). Identification of SUMO-2/3-modified proteins associated with mitotic chromosomes. Proteomics.

[153] Edelmann M.J., Shack L.A., Naske C.D., Walters K.B., Nanduri B. (2014). SILAC-Based Quantitative Proteomic Analysis of Human Lung Cell Response to Copper Oxide Nanoparticles. PLoS ONE.

[154] Chen X., Wei S., Ji Y., Guo X., Yang F. (2015). Quantitative proteomics using SILAC: Principles, applications, and developments. Proteomics.

[155] Fredriksson S., Gullberg M., Jarvius J., Olsson C., Pietras K., Gústafsdóttir S.M., Östman A., Landegren U. (2002). Protein detection using proximity-dependent DNA ligation assays. Nat. Biotechnol..

[156] Akter S., Fu L., Jung Y., Conte M.L., Lawson J.R., Lowther W.T., Sun R., Liu K., Yang J., Carroll K.S. (2018). Chemical proteomics reveals new targets of cysteine sulfinic acid reductase. Nat. Chem. Biol..

[157] Poole L.B. (2015). The basics of thiols and cysteines in redox biology and chemistry. Free. Radic. Biol. Med..

[158] Brandi J., Noberini R., Bonaldi T., Cecconi D. (2022). Advances in enrichment methods for mass spectrometry-based proteomics analysis of post-translational modifications. J. Chromatogr. A.

[159] Ren R.J., Dammer E.B., Wang G., Seyfried N.T., Levey A.I. (2014). Proteomics of protein post-translational modifications implicated in neurodegeneration. Transl. Neurodegener..

[160] Humphrey S.J., James D.E., Mann M. (2015). Protein phosphorylation: A major switch mechanism for metabolic regulation. Trends Endocrinol. Metab..

[161] Rani N., Sahu M., Ambasta R.K., Kumar P. (2023). Triaging between post-translational modification of cell cycle regulators and their therapeutics in neurodegenerative diseases. Ageing Res. Rev..

[162] Kurtishi A., Rosen B., Patil K.S., Alves G.W., Møller S.G. (2019). Cellular proteostasis in neurodegeneration. Mol. Neurobiol..

[163] Jean Beltran P.M., Federspiel J.D., Sheng X., Cristea I.M. (2017). Proteomics and integrative omic approaches for understanding host–pathogen interactions and infectious diseases. Mol. Syst. Biol..

[164] Beck H.C., Nielsen E.C., Matthiesen R., Jensen L.H., Sehested M., Finn P., Grauslund M., Hansen A.M., Jensen O.N. (2006). Quantitative proteomic analysis of post-translational modifications of human histones. Mol. Cell Proteom..

[165] Dekker F.J., van den Bosch T., Martin N.I. (2014). Small molecule inhibitors of histone acetyltransferases and deacetylases are po-tential drugs for inflammatory diseases. Drug Discov. Today.

[166] Gupta R., Kumar P. (2021). Computational Analysis Indicates That PARP1 Acts as a Histone Deacetylases Interactor Sharing Common Lysine Residues for Acetylation, Ubiquitination, and SUMOylation in Alzheimer’s and Parkinson’s Disease. ACS Omega.

[167] Zhang S., Meng Y., Zhou L., Qiu L., Wang H., Su D., Zhang B., Chan K., Han J. (2022). Targeting epigenetic regulators for inflammation: Mechanisms and intervention therapy. MedComm.

[168] Xu L., Feng J., Tang H., Dong Y., Shu M., Chen X. (2020). Chidamide epigenetically represses autophagy and exerts co-operative antimyeloma activity with bortezomib. Cell Death Dis..

[169] Rastgoo N., Pourabdollah M., Abdi J., Reece D., Chang H. (2018). Dysregulation of EZH2/miR-138 axis contributes to drug resistance in multiple myeloma by downregulating RBPMS. Leukemia.

[170] Nara M., Teshima K., Watanabe A., Ito M., Iwamoto K., Kitabayashi A., Kume M., Hatano Y., Takahashi N., Iida S. (2013). Bortezomib Reduces the Tumorigenicity of Multiple Myeloma via Downregulation of Upregulated Targets in Clon-ogenic Side Population Cells. PLoS ONE.

[171] Zhou L., Zhang Q., Zhang P., Sun L., Peng C., Yuan Z., Cheng J. (2017). c-Abl-mediated Drp1 phosphorylation promotes oxidative stress-induced mitochondrial fragmentation and neuronal cell death. Cell Death Dis..

[172] Lan W., Santofimia-Castaño P., Swayden M., Xia Y., Zhou Z., Audebert S., Camoin L., Huang C., Peng L., Jiménez-Alesanco A. (2020). ZZW-115–dependent inhibition of NUPR1 nuclear translocation sensitizes cancer cells to genotoxic agents. J. Clin. Investig..

[173] Santofimia-Castaño P., Lan W., Bintz J., Gayet O., Carrier A., Lomberk G., Neira J.L., González A., Urrutia R., Soubeyran P. (2018). Inactivation of NUPR1 promotes cell death by coupling ER-stress responses with necrosis. Sci. Rep..

[174] Santofimia-Castaño P., Xia Y., Lan W., Zhou Z., Huang C., Peng L., Soubeyran P., Velázquez-Campoy A., Abián O., Rizzuti B. (2019). Ligand-based design identifies a potent NUPR1 inhibitor exerting anticancer activity via necroptosis. J. Clin. Investig..

[175] Tenreiro S., Eckermann K., Outeiro T.F. (2014). Protein phosphorylation in neurodegeneration: Friend or foe?. Front. Mol. Neurosci..

[176] Rasmi Y., Shokati A., Hassan A., Aziz S.G.-G., Bastani S., Jalali L., Moradi F., Alipour S. (2023). The role of DNA methylation in progression of neurological disorders and neurodegenerative diseases as well as the prospect of using DNA methylation inhibitors as therapeutic agents for such disorders. IBRO Neurosci. Rep..

[177] Kawahata I., Finkelstein D.I., Fukunaga K. (2022). Pathogenic Impact of α-Synuclein Phosphorylation and Its Kinases in α-Synucleinopathies. Int. J. Mol. Sci..

[178] Mandel N., Agarwal N. (2022). Role of SUMOylation in Neurodegenerative Diseases. Cells.

[179] Schmidt M.F., Gan Z.Y., Komander D., Dewson G. (2021). Ubiquitin signalling in neurodegeneration: Mechanisms and therapeutic opportunities. Cell Death Differ..

[180] Nakamura T., Oh C.-K., Zhang X., Lipton S.A. (2021). Protein S-nitrosylation and oxidation contribute to protein misfolding in neurodegeneration. Free. Radic. Biol. Med..

[181] Tsoi P.S., Quan M.D., Ferreon J.C., Ferreon A.C.M. (2023). Aggregation of Disordered Proteins Associated with Neurodegeneration. Int. J. Mol. Sci..

[182] Lee B.E., Suh P.-G., Kim J.-I. (2021). O-GlcNAcylation in health and neurodegenerative diseases. Exp. Mol. Med..

[183] Tabrez S., Jabir N.R., Shakil S., Greig N.H., Alam Q., Abuzenadah A.M., Damanhouri G.A., Kamal M.A. (2012). A Synopsis on the Role of Tyrosine Hydroxylase in Parkinson’s Disease. CNS Neurol. Disord.-Drug Targets.

[184] Yamada M., Hamaguchi T. (2018). The sulfation code for propagation of neurodegeneration. J. Biol. Chem..

